# Supplementary material for: Steroid Metabolome Analysis in Dichorionic Diamniotic Twin Pregnancy
Source: Int J Mol Sci. 2024 Jan 27;25(3):1591. doi: 10.3390/ijms25031591 (PMC10855299; doi:10.3390/ijms25031591)
Supplement: Supplementary file 1 [file ijms-25-01591-s001.zip › ijms-2773599-supplementary/Table Suppement 9.pdf]

International Journal of *Molecular Sciences*

MDPI

Supplementary Table 9. Power calculation for the study

We tested the required number of measurements to achieve a minimum test power of 0.8 at the significance level of a non-parametric Mann-Whitney U test of p<0. 05 and we found a minimum number of measurements in the groups of n=15 at equivalent number of measurements and this number was achieved (maternal blood n=16 for controls and n=18 for twin pregnancies). We further assumed a number of n=15+ 2×15 = 30 measurements in the case of cord blood and umbilical artery and umbilical vein blood i.e. an allocation ratio of 1:2. For this ratio, the numbers n1=11 for the less numerous group (maternal blood) and n2=22 for the more numerous groups were determined. These counts were exceeded for both maternal blood and arterial cord blood (n=16 for controls, n=27 for twin pregnancies) and venous cord blood (n=16 for controls, n=37 for twin pregnancies). These data indicate that there were no insufficient measurements in either group in terms of the required statistical power of the test. (Sample size calculator Version 1.061, Contact: robin.ristl@univie.ac.at, https://homepage.univie.ac.at/robin.ristl/samplesize.php?test=wilcoxon).

PregC_A

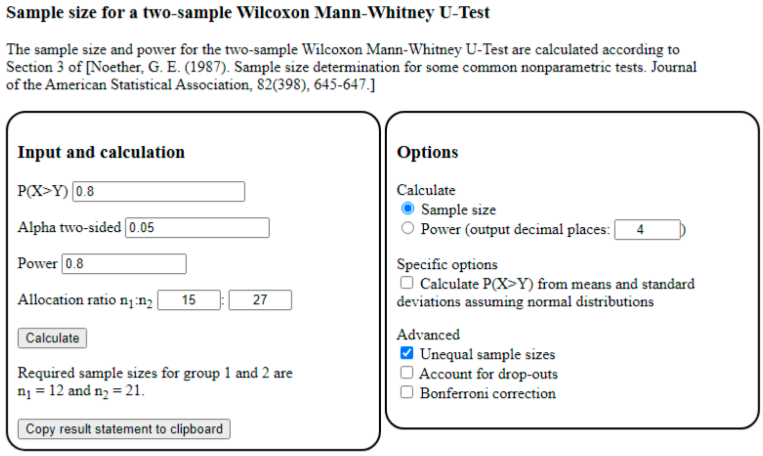

PregC_V

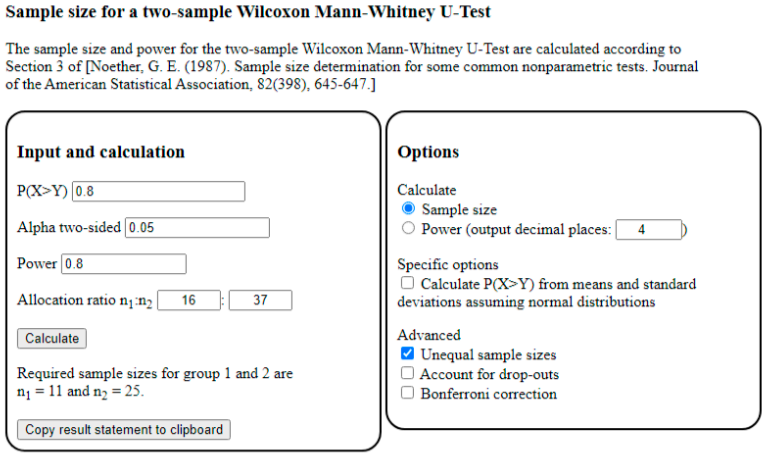

PregC_M

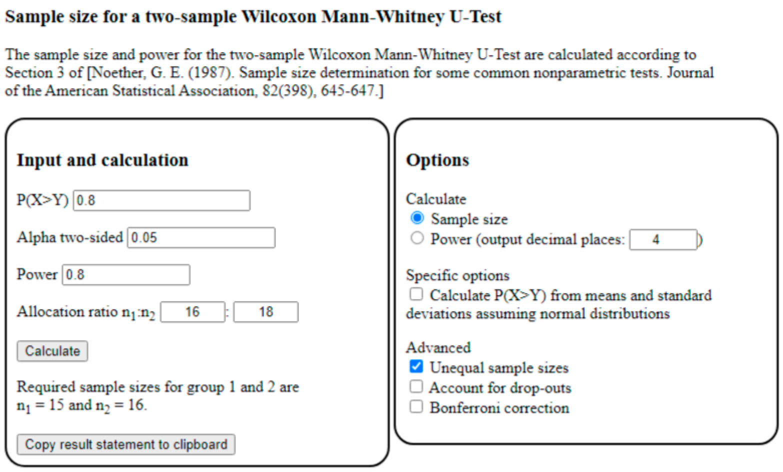

Sample size for a two-sample Wilcoxon Mann-Whitney U-Test

The sample size and power for the two-sample Wilcoxon Mann-Whitney U-Test are calculated according to Section 3 of [Noether, G. E. (1987). Sample size determination for some common nonparametric tests. Journal of the American Statistical Association, 82(398), 645-647.]

Input and calculation

P(X>Y) 0.8

Alpha two-sided 0.05

Power 0.8

Allocation ratio n_1:n_2 16 : 18

Calculate

Required sample sizes for group 1 and 2 are $n_1 = 15$ and $n_2 = 16$.

Copy result statement to clipboard

Options

Calculate
- Sample size
- Power (output decimal places: 4)

Specific options
- Calculate P(X>Y) from means and standard deviations assuming normal distributions

Advanced
- Unequal sample sizes
- Account for drop-outs
- Bonferroni correction
