# Supplementary material for: Steroid Metabolome Analysis in Dichorionic Diamniotic Twin Pregnancy
Source: Int J Mol Sci. 2024 Jan 27;25(3):1591. doi: 10.3390/ijms25031591 (PMC10855299; doi:10.3390/ijms25031591)
Supplement: Supplementary file 1 [file ijms-25-01591-s001.zip › ijms-2773599-supplementary/Table Supplement 1.pdf]

International Journal of *Molecular Sciences*

MDPI

Supplementary Table 1. Relationships between twin, diamniotic, dichorionic pregnancy (vs. singleton pregnancy) and relevant parameters (with significant variable importance, p<0.05) in serum from umbilical artery at labour as evaluated by OPLS and ordinary multiple regression (OMR) models (for details see Statistical analysis)

		**OPLS, predictive component**							**Multiple regression**		
	**Variable**	Variable importance	t-statistics		Component loading	t-statistics	R[a]		Regression coefficient	t-statistics	
EXPLAINING VARIABLES	Gestational age	1.21	5.02	**	-0.190	-6.29	-0.584	**	-0.102	-3.52	**
	Pregnenolone	0.918	3.38	**	0.162	3.55	0.500	**	0.077	2.93	*
	Pregnenolone, C	1.032	2.38	*	-0.187	-2.51	-0.577	*	-0.099	-3.49	**
	16α-Hydroxypregnenolone	1.225	3.88	**	0.211	3.89	0.648	**	0.054	1.64	
	20α-Dihydropregnenolone	1.27	4.14	**	0.209	5.04	0.644	**	0.091	3.43	**
	Dehydroepiandrosterone, C	0.994	3.67	**	-0.182	-4.07	-0.562	**	-0.098	-5.25	**
	7-oxo-DHEA	0.86	3.08	**	0.135	3.08	0.409	**	0.010	0.29	
	Androstenediol, C	0.62	2.51	*	-0.107	-3.50	-0.331	**	-0.027	-0.88	
	17-Hydroxyprogesterone	0.455	2.52	*	0.065	1.94	0.200	*	0.016	0.57	
	17,20α-Dihydroxy-4-pregnene-3-one	0.623	2.26	*	0.084	2.00	0.257	*	0.017	0.54	
	20α-Dihydroprogesterone	0.945	3.02	**	0.175	4.00	0.536	**	0.004	0.14	
	Estradiol, C	0.852	2.75	*	0.173	2.95	0.539	*	0.022	0.68	
	5α-Dihydroprogesterone	1.248	4.70	**	0.207	5.55	0.634	**	0.051	1.27	
	Allopregnanolone	1.131	9.26	**	0.200	6.40	0.614	**	0.035	2.40	*
	Allopregnanolone, C	0.662	2.04	*	0.114	2.30	0.359	*	-0.009	-0.40	
	Isopregnanolone	1.511	12.29	**	0.260	23.47	0.798	**	0.063	2.98	*
	5α,20α-Tetrahydroprogesterone	1.309	7.94	**	0.235	7.49	0.722	**	0.034	2.15	*
	5α-Pregnane-3α,20α-diol	1.267	8.12	**	0.218	6.34	0.669	**	0.049	3.66	**
	5α-Pregnane-3α,20α-diol, C	0.791	2.49	*	0.144	2.79	0.449	*	0.019	0.73	
	5α-Pregnane-3β,20α-diol	1.525	9.48	**	0.275	10.89	0.844	**	0.063	2.91	*
	5α-Pregnane-3β,20α-diol, C	0.907	3.29	**	0.152	3.18	0.475	**	0.023	1.14	
	17-Hydroxyallopregnanolone	0.854	3.48	**	0.147	3.67	0.450	**	0.011	0.45	
	17-Hydroxyallopregnanolone, C	0.506	2.58	*	0.078	2.33	0.236	*	-0.031	-2.09	*
	17-Hydroxypregnanolone	0.678	2.88	*	0.093	2.93	0.283	*	0.021	0.54	
	17-Hydroxypregnanolone, C	0.812	2.97	*	0.110	1.87	0.339		0.028	1.47	
	5α-Pregnane-3α,17,20α-triol	0.678	2.54	*	0.075	1.54	0.229		0.024	1.25	
	Androsterone, C	0.793	1.95	*	-0.158	-2.27	-0.486	*	-0.094	-4.03	**
	5α-Androstane-3α,17β-diol	0.777	1.95	*	0.114	1.46	0.345		0.042	1.62	
	5α-Androstane-3α,17β-diol, C	0.709	2.03	*	0.094	1.42	0.295		0.020	0.70	
	Corticosterone	0.662	2.77	*	0.089	1.87	0.271		0.012	0.54	
	21-Deoxycortisol	1.138	4.36	**	0.175	4.05	0.537	**	0.047	1.97	*
	11-Deoxycorticosterone	0.897	4.06	**	0.144	3.59	0.441	**	0.037	1.53	
	11β-Hydroxytestosterone, C	1.161	3.06	**	-0.175	-2.47	-0.539	*	-0.101	-3.09	**
	3α,5β-Tetrahydrocorticosterone	1.796	11.82	**	0.291	13.01	0.906	**	0.117	5.94	**
	11β-Hydroxyepiandrosterone	0.625	2.63	*	0.125	3.38	0.379	**	-0.023	-1.03	
	11β-Hydroxyepiandrosterone, C	0.855	3.20	**	0.109	2.10	0.351	*	0.046	1.27	
	Twins (diamniotic dichorionic)				1.000	25.93	0.862	**			
	Explained variability = 74.3% (63.1% after cross-validation), Sensitivity = 0.962(0.888-1), Specificity = 1(1-1)										

[a]*R…Component loading expressed as a correlation coefficient with predictive component, *p<0.05, **p<0.01, LLR…logarithm of likelihood ratio (the ratio of the probability that the phenomenon occurs - twin pregnancy) to the probability that the phenomenon does not occur - singleton pregnancy), C…conjugated steroid*
