# Supplementary material for: Steroid Metabolome Analysis in Dichorionic Diamniotic Twin Pregnancy
Source: Int J Mol Sci. 2024 Jan 27;25(3):1591. doi: 10.3390/ijms25031591 (PMC10855299; doi:10.3390/ijms25031591)
Supplement: Supplementary file 1 [file ijms-25-01591-s001.zip › ijms-2773599-supplementary/Table Supplement 2.pdf]

International Journal of
Molecular Sciences

Supplementary Table 2. Relationships between twin, diamniotic, dichorionic pregnancy (vs. singleton pregnancy) and relevant parameters (with significant variable importance, p<0.05) in serum from umbilical vein at labour as evaluated by OPLS and ordinary multiple regression (OMR) models (for details see Statistical analysis)

		OPLS, predictive component							Multiple regression		
	**Variable**	Variable importance	t-statistics		Component loading	t-statistics	R^a		Regression coefficient	t-statistics	
EXPLAINING VARIABLES	Gestational age	1.149	4.13	**	-0.173	-3.82	-0.507	**	-0.062	-3.41	**
	Pregnenolone, C	1.032	4.03	**	-0.235	-6.58	-0.686	**	-0.056	-3.60	**
	17-Hydroxypregnenolone, C	0.465	3.46	**	-0.149	-3.32	-0.441	**	-0.025	-3.64	**
	16α-Hydroxypregnenolone	0.924	5.93	**	0.156	3.28	0.456	**	0.050	6.06	**
	20α-Dihydropregnenolone, C	0.748	4.18	**	-0.207	-5.40	-0.607	**	-0.041	-4.21	**
	Dehydroepiandrosterone, C	1.061	3.65	**	-0.220	-3.81	-0.643	**	-0.058	-3.34	**
	5-Androstene-3β,16α,17β-triol, C	0.811	3.99	**	-0.229	-4.52	-0.669	**	-0.044	-4.94	**
	20α-Dihydroprogesterone	0.855	3.41	**	0.208	4.66	0.608	**	0.046	3.14	**
	Epitestosterone, C	0.67	3.60	**	-0.157	-3.82	-0.459	**	-0.036	-4.41	**
	5α-Dihydroprogesterone	1.446	8.30	**	0.283	7.78	0.829	**	0.079	6.01	**
	Allopregnanolone	1.163	7.63	**	0.277	14.49	0.811	**	0.063	7.65	**
	Isopregnanolone	1.464	10.67	**	0.293	15.74	0.858	**	0.080	7.50	**
	Epipregnanolone	0.713	2.86	*	0.137	3.65	0.399	**	0.039	2.82	*
	5α,20α-Tetrahydroprogesterone	1.275	19.49	**	0.275	12.21	0.804	**	0.069	9.69	**
	5α-Pregnane-3α,20α-diol	1.101	6.52	**	0.261	18.31	0.765	**	0.060	7.03	**
	5α-Pregnane-3β,20α-diol	1.328	10.23	**	0.278	19.85	0.815	**	0.072	8.05	**
	5β,20α-Tetrahydroprogesterone	0.664	3.09	**	0.112	3.05	0.326	**	0.036	2.55	*
	5β-Pregnane-3β,20α-diol	0.552	4.37	**	0.114	5.48	0.332	**	0.030	4.06	**
	17-Hydroxyallopregnanolone	0.711	3.10	**	0.187	8.99	0.546	**	0.039	3.66	**
	Epiandrosterone, C	0.923	3.46	**	-0.174	-3.63	-0.509	**	-0.050	-3.14	**
	11-Deoxycorticosterone	0.811	9.37	**	0.119	3.36	0.350	**	0.044	7.04	**
	3α,5β-Tetrahydrocorticosterone	1.426	11.13	**	0.242	7.01	0.700	**	0.078	6.30	**
	11β-Hydroxyepiandrosterone	0.729	3.65	**	0.203	7.56	0.595	**	0.040	3.78	**
	Twins (diamniotic dichorionic)				1.000	19.20	0.764	**			
	Explained variability = 58.3% (55.3% after cross-validation), Sensitivity = 0.943(0.866-1), Specificity = 0.8(0.598-1)										

[a]*R…Component loading expressed as a correlation coefficient with predictive component, *p<0.05, **p<0.01, LLR…logarithm of likelihood ratio (the ratio of the probability that the phenomenon occurs - twin pregnancy) to the probability that the phenomenon does not occur - singleton pregnancy), C…conjugated steroid*
