# Supplementary material for: Steroid Metabolome Analysis in Dichorionic Diamniotic Twin Pregnancy
Source: Int J Mol Sci. 2024 Jan 27;25(3):1591. doi: 10.3390/ijms25031591 (PMC10855299; doi:10.3390/ijms25031591)
Supplement: Supplementary file 1 [file ijms-25-01591-s001.zip › ijms-2773599-supplementary/Table Supplement 3.pdf]

International Journal of *Molecular Sciences*

MDPI

Supplementary Table 3. Relationships between twin, diamniotic, dichorionic pregnancy (vs. singleton pregnancy) and relevant parameters (with significant variable importance, p<0.05) in maternal serum at labour as evaluated by OPLS and ordinary multiple regression (OMR) models (for details see Statistical analysis)

EXPLAINING VARIABLES

	OPLS, predictive component							Multiple regression		
**Variable**	Variable importance	t-statistics		Component loading	t-statistics	R^a		Regression coefficient	t-statistics	
Number of male fetuses	1.033	7.39	**	0.115	9.56	0.524	**	0.079	3.85	**
Gestational age	1.025	3.33	**	-0.133	-3.37	-0.609	**	-0.054	-1.94	*
Pregnenolone	1.564	10.74	**	0.197	12.68	0.903	**	0.083	4.14	**
17-Hydroxypregnenolone	0.843	2.18	*	0.121	2.73	0.552	*	0.030	0.85	
17-Hydroxypregnenolone, C	0.585	2.27	*	0.072	2.10	0.328	*	0.011	0.41	
16α-Hydroxypregnenolone	1.327	7.77	**	0.165	7.95	0.756	**	0.024	1.06	
20α-Dihydropregnenolone	1.367	7.63	**	0.182	7.38	0.831	**	0.081	6.50	**
Dehydroepiandrosterone	0.74	2.49	*	0.113	2.89	0.517	*	0.041	1.43	
7-oxo-DHEA	1.294	6.11	**	0.164	6.25	0.751	**	0.041	2.34	*
7β-Hydroxy-DHEA	0.777	2.62	*	0.112	2.78	0.511	*	0.015	0.45	
5-Androstene-3β,16α,17β-triol, C	1.152	4.51	**	0.148	3.95	0.675	**	0.041	1.55	
Progesterone	0.619	1.97	*	0.092	2.46	0.423	*	0.025	1.73	
17-Hydroxyprogesterone	1.152	3.59	**	0.145	3.50	0.663	**	0.018	1.13	
17,20α-Dihydroxy-4-pregnene-3-one	1.265	6.80	**	0.163	6.62	0.747	**	0.021	1.21	
16α-Hydroxyprogesterone	1.049	4.56	**	0.123	3.99	0.565	**	0.020	0.76	
20α-Dihydroprogesterone	1.462	12.81	**	0.192	14.60	0.877	**	0.053	4.19	**
20α-Dihydroprogesterone, C	1.155	6.43	**	0.141	5.45	0.644	**	0.036	1.65	
16α-Hydroxytestosterone	0.749	3.04	**	0.090	2.73	0.413	*	-0.014	-0.38	
16α-Hydroxytestosterone, C	0.902	4.14	**	0.104	4.04	0.477	**	0.010	0.57	
Epitestosterone, C	0.712	2.47	*	0.103	3.28	0.470	**	-0.011	-0.51	
5α-Dihydrotestosterone	0.329	3.00	*	0.044	1.68	0.203		-0.038	-1.66	
Estrone	0.621	2.07	*	0.091	3.41	0.416	**	-0.005	-0.17	
Estrone, C	0.653	2.67	*	0.076	2.59	0.350	*	0.004	0.20	
Estradiol	0.973	5.44	**	0.123	5.85	0.567	**	0.013	0.88	
Estradiol, C	0.75	2.01	*	0.098	1.63	0.448		0.015	0.66	
Estriol	0.598	2.67	*	0.065	2.06	0.297	*	0.008	0.29	
5α-Dihydroprogesterone	1.118	7.77	**	0.140	6.06	0.639	**	0.010	0.79	
Allopregnanolone	1.015	4.26	**	0.136	3.84	0.622	**	0.007	0.44	
Isopregnanolone	1.526	10.91	**	0.184	9.01	0.839	**	0.056	4.12	**
Isopregnanolone, C	0.582	3.59	**	0.074	3.61	0.340	**	-0.035	-3.68	**
5β-Dihydroprogesterone	0.983	3.83	**	0.124	3.22	0.569	**	0.019	1.06	
Pregnanolone	0.795	2.57	*	0.108	2.59	0.493	*	0.008	0.41	
Epipregnanolone	0.825	2.72	*	0.120	3.50	0.550	**	0.003	0.15	
Epipregnanolone, C	0.639	5.85	**	0.080	6.11	0.365	**	-0.031	-2.54	*
5α,20α-Tetrahydroprogesterone	1.4	12.97	**	0.176	11.29	0.804	**	0.037	3.53	**
5α,20α-Tetrahydroprogesterone, C	0.522	2.09	*	0.065	1.66	0.299		-0.011	-0.51	
5α-Pregnane-3α,20α-diol	1.415	9.31	**	0.182	9.53	0.832	**	0.049	5.88	**
5α-Pregnane-3β,20α-diol	1.592	8.96	**	0.193	7.55	0.884	**	0.075	4.73	**
5α-Pregnane-3β,20α-diol, C	0.549	2.00	*	0.064	1.59	0.292		0.016	0.73	
5β,20α-Tetrahydroprogesterone	1.273	8.74	**	0.166	7.42	0.757	**	0.049	4.10	**
5β-Pregnane-3α,20α-diol	1.286	6.95	**	0.170	7.57	0.779	**	0.060	4.53	**
5β-Pregnane-3β,20α-diol	0.953	5.68	**	0.134	5.78	0.614	**	0.053	5.01	**
17-Hydroxyallopregnanolone	1	4.14	**	0.125	5.02	0.574	**	-0.003	-0.10	
17-Hydroxyallopregnanolone, C	0.943	9.01	**	0.109	9.37	0.498	**	-0.008	-0.80	
17-Hydroxypregnanolone	0.999	3.63	**	0.118	3.43	0.541	**	0.016	0.67	
17-Hydroxypregnanolone, C	0.969	4.84	**	0.128	4.95	0.587	**	0.017	1.12	
5α-Pregnane-3α,17,20α-triol	0.884	5.82	**	0.117	6.89	0.536	**	-0.011	-0.90	
5β-Pregnane-3α,17,20α-triol	0.742	4.68	**	0.099	4.97	0.455	**	0.008	0.39	
5α-Androstane-3,17-dione	0.586	3.08	**	0.067	2.37	0.308	*	-0.004	-0.21	

Androsterone	0.516	2.13	*	0.063	1.52	0.286		-0.005	-0.26	
Epiandrosterone	1.099	4.57	**	0.143	4.28	0.652	**	0.050	2.45	*
Etiocholanolone	0.312	2.19	*	0.052	2.67	0.239	*	-0.002	-0.06	
5α-Androstane-3α,17β-diol	0.735	2.67	*	0.093	2.32	0.425	*	0.051	1.92	*
11-Deoxycortisol	0.798	2.96	*	0.115	3.20	0.526	**	-0.009	-0.39	
11-Deoxycorticosterone	1.304	6.05	**	0.161	7.21	0.766	**	0.034	1.61	
11-Deoxycorticosterone, C	0.801	2.52	*	0.086	2.36	0.395	*	0.009	0.40	
11β-Hydroxytestosterone	1.166	11.45	**	0.146	9.51	0.711	**	0.014	1.27	
11β-Hydroxytestosterone, C	0.723	3.24	**	0.082	3.42	0.400	**	0.003	0.18	
3α,5β-Tetrahydrocorticosterone	1.441	10.92	**	0.171	12.75	0.807	**	0.047	3.99	**
11β-Hydroxyandrosterone	0.872	3.12	**	0.114	3.68	0.560	**	0.000	0.02	
11β-Hydroxyepiandrosterone	1.262	5.77	**	0.163	5.25	0.784	**	0.019	1.74	
11β-Hydroxyepiandrosterone, C	0.505	2.23	*	0.053	1.50	0.236		0.000	-0.01	
Twins (diamniotic dichorionic), LLR				1.000	41.32	0.917	**			
Explained variability = 84% (68.8% after cross-validation), Sensitivity = 1(1-1), Specificity = 1(1-1)										

*[a]R…Component loading expressed as a correlation coefficient with predictive component, *p<0.05, **p<0.01, LLR…logarithm of likelihood ratio (the ratio of the probability that the phenomenon occurs - twin pregnancy) to the probability that the phenomenon does not occur - singleton pregnancy), C…conjugated steroid*

2
