# Supplementary material for: Steroid Metabolome Analysis in Dichorionic Diamniotic Twin Pregnancy
Source: Int J Mol Sci. 2024 Jan 27;25(3):1591. doi: 10.3390/ijms25031591 (PMC10855299; doi:10.3390/ijms25031591)
Supplement: Supplementary file 1 [file ijms-25-01591-s001.zip › ijms-2773599-supplementary/Table Supplement 4.pdf]

International Journal of
Molecular Sciences

MDPI

Supplementary Table 4. Steroid differences between female and male foetuses in umbilical arterial blood

Steroid	Female	Male	p	η_p^2
5-Androstene-3β,16α,17β-triol sulfate [nM]	461 (404, 527)	629 (541, 736)	0.039	0.113
17,20α-Dihydroxy-4-pregnene-3-one [nM]	13.4 (11.3, 15.9)	8.59 (7.14, 10.4)	0.021	0.137
Androstenedione [nM]	3.1 (2.67, 3.61)	2.19 (1.86, 2.58)	0.036	0.116
Testosterone [pM]	92 (45.8, 173)	705 (354, 1430)	0.005	0.198
16α-Hydroxytestosterone [nM]	12.9 (10.6, 15.6)	7.51 (5.95, 9.39)	0.017	0.148
5α-Dihydrotestosterone [pM]	35.9 (22.2, 56.6)	98.4 (59.9, 161)	0.046	0.103
5β-Pregnane-3α,17,20α-triol [nM]	2.34 (2.09, 2.63)	1.46 (1.3, 1.65)	<0.001	0.291
5β-Pregnane-3α,17,20α-triol, conjugated [nM]	592 (476, 751)	307 (249, 384)	0.007	0.184
Etiocholanolone [pM]	55 (47.8, 62.8)	30.7 (25.1, 37)	0.001	0.25
11β-Hydroxyandrostenedione [nM]	9.67 (8.02, 11.7)	6.14 (4.99, 7.56)	0.031	0.12
3α,5β-Tetrahydrocorticosterone [nM]	2.13 (1.65, 2.72)	1.13 (0.841, 1.49)	0.025	0.139

The differences between twin and singleton pregnancies for each steroid were evaluated using a linear model consisting of factors Pregnancy type (Twin vs. Singleton) and Gender (Male vs. Female) adjusted for maternal age and gestational age at labour. Significant differences (p<0.05) are in bold, p…p-value, ηp2…effect size (0.01 ~ small, 0.06 ~ medium, >0.14 ~ large)
