# Supplementary material for: Steroid Metabolome Analysis in Dichorionic Diamniotic Twin Pregnancy
Source: Int J Mol Sci. 2024 Jan 27;25(3):1591. doi: 10.3390/ijms25031591 (PMC10855299; doi:10.3390/ijms25031591)
Supplement: Supplementary file 1 [file ijms-25-01591-s001.zip › ijms-2773599-supplementary/Table Supplement 5.pdf]

International Journal of
Molecular Sciences

MDPI

Supplementary Table 5. Steroid differences between female and male foetuses in umbilical venous blood

Steroid	Female	Male	*p*	η_p^2
5-Androstene-3β,16α,17β-triol sulfate [nM]	414 (359, 476)	669 (583, 768)	0.001	0.209
Testosterone [pM]	28.1 (17.8, 43.5)	115 (71.9, 194)	0.003	0.175
Estrone [nM]	54.6 (44.5, 65.7)	27.7 (20.2, 36.3)	0.006	0.146
5β-Pregnane-3α,17,20α-triol [nM]	2.21 (1.96, 2.48)	1.51 (1.32, 1.72)	0.004	0.171
5β-Pregnane-3α,17,20α-triol, conjugated [nM]	667 (505, 898)	300 (233, 390)	0.005	0.154
5α-Androstane-3,17-dione [nM]	0.224 (0.189, 0.27)	0.156 (0.134, 0.184)	0.034	0.092
Androsterone [nM]	144 (121, 173)	91.3 (78.6, 107)	0.007	0.148
Etiocholanolone [pM]	54.6 (47.1, 63.5)	36.6 (31.6, 42.5)	0.01	0.137
5α-Androstane-3β,17β-diol,conjugated [nM]	5.46 (4.67, 6.38)	7.64 (6.5, 8.97)	0.04	0.086
Cortisol [nM]	129 (117, 144)	100 (89.6, 112)	0.024	0.103
Cortisone [nM]	200 (179, 224)	160 (143, 179)	0.048	0.079
11β-Hydroxyandrostenedione [nM]	6.65 (5.5, 8.05)	4.5 (3.72, 5.45)	0.044	0.083

The differences between twin and singleton pregnancies for each steroid were evaluated using a linear model consisting of factors Pregnancy type (Twin vs. Singleton) and Gender (Male vs. Female) adjusted for maternal age and gestational age at labour. Significant differences ($p<0.05$) are in bold, p…p-value, ηp2…effect size (0.01 ~ small, 0.06 ~ medium, >0.14 ~ large)
