# Supplementary material for: Steroid Metabolome Analysis in Dichorionic Diamniotic Twin Pregnancy
Source: Int J Mol Sci. 2024 Jan 27;25(3):1591. doi: 10.3390/ijms25031591 (PMC10855299; doi:10.3390/ijms25031591)
Supplement: Supplementary file 1 [file ijms-25-01591-s001.zip › ijms-2773599-supplementary/Table Supplement 6.pdf]

International Journal of *Molecular Sciences*

MDPI

Supplementary Table 6. Steroid differences between female and male foetuses in maternal venous blood

**Steroid**	**Female**	**Male**	*p*	η_p^2
5-Androstene-3β,7β,17β-triol [pM]	207 (143, 297)	56.6 (33.8, 91)	0.006	0.238
16α-Hydroxyprogesterone [nM]	28.2 (24.4, 32.7)	42.1 (34.5, 52.2)	0.037	0.147
Estrone [nM]	28 (22.4, 35.1)	14.8 (11.3, 19.2)	0.017	0.18
Estrone sulfate [nM]	258 (201, 324)	447 (354, 554)	0.031	0.155
Estradiol [nM]	113 (98.8, 129)	75.4 (64.6, 88)	0.012	0.203
Epipregnanolone, conjugated [nM]	186 (151, 229)	349 (266, 462)	0.019	0.183
5β-Pregnane-3α,17,20α-triol [nM]	6.91 (5.93, 8.04)	4.37 (3.56, 5.31)	0.017	0.18
5β-Pregnane-3α,17,20α-triol, conjugated [nM]	562 (462, 693)	304 (246, 378)	0.007	0.184
Androsterone [nM]	0.696 (0.575, 0.855)	0.441 (0.36, 0.546)	0.04	0.138
Androsterone sulfate [nM]	321 (252, 408)	169 (122, 229)	0.031	0.155
Epiandrosterone [nM]	0.411 (0.317, 0.536)	0.19 (0.14, 0.257)	0.014	0.196
Epiandrosterone sulfate [nM]	86 (70.1, 106)	53 (41.9, 67)	0.043	0.139
Etiocholanolone [pM]	185 (160, 215)	131 (110, 156)	0.049	0.127
5α-Androstane-3α,17β-diol [pM]	98.5 (78.7, 126)	59.6 (47.7, 75.7)	0.05	0.131
11β-Hydroxyandrosterone [pM]	195 (162, 235)	312 (248, 395)	0.043	0.144

The differences between twin and singleton pregnancies for each steroid were evaluated using a linear model consisting of factors Pregnancy type (Twin vs. Singleton) and Gender (Male vs. Female) adjusted for maternal age and gestational age at labour. Significant differences ($p<0.05$) are in bold, p…p-value, ηp2…effect size (0.01 ~ small, 0.06 ~ medium, >0.14 ~ large)
