# Supplementary material for: Steroid Metabolome Analysis in Dichorionic Diamniotic Twin Pregnancy
Source: Int J Mol Sci. 2024 Jan 27;25(3):1591. doi: 10.3390/ijms25031591 (PMC10855299; doi:10.3390/ijms25031591)
Supplement: Supplementary file 1 [file ijms-25-01591-s001.zip › ijms-2773599-supplementary/Table Supplement 7.pdf]

International Journal of
Molecular Sciences

MDPI

Supplementary Table 7. List of steroid metabolites analysed, according to Hill, M.; Hána, V., Jr.; Velíková, M.; Pařízek, A.; Kolátorová, L.; Vítků, J.; Škodová, T.; Šimková, M.; Šimják, P.; Kancheva, R.; et al. A method for determination of one hundred endogenous steroids in human serum by gas chromatography-tandem mass spectrometry. *Physiol Res* **2019**, *68*, 179–207.

Abbreviation	Steroid analyzed
Preg	Pregnenolone
Preg17	17α-Hydroxypregnenolone
Preg16a	16α-Hydroxypregnenolone
Preg20a	20α-Dihydropregnenolone
DHEA	Dehydroepiandrosterone
DHEA7a	7α-Hydroxy-DHEA
DHEA7o	7-oxo-DHEA
DHEA7b	7β-Hydroxy-DHEA
Adiol	5-Androstene-3β, 17β-diol
AT7a	5-Androstene-3β,7α,17β-triol
AT7b	5-Androstene-3β,7β,17β-triol
AT16a	5-Androstene-3β,16α,17β-triol
Prog	Progesterone
Prog17	17α-Hydroxyprogesterone
Prog17a20a	17α,20α-Dihydroxy-4-pregnene-3-one
Prog16a	16α-Hydroxyprogesterone
Prog20a	20α-Dihydroprogesterone
Adion	Androstenedione
T	Testosterone
T16a	16α-Hydroxytestosterone
DHT5a	5α-Dihydrotestosterone
E1	Estrone
E2	Estradiol
E3	Estriol
P5a	5α-Dihydroprogesterone
P3a5a	Allopregnanolone
P3b5a	Isopregnanolone
P5b	5β-Dihydroprogesterone
P3a5b	Pregnanolone
P3b5b	Epipregnanolone
P5a20a	5α,20α-Tetrahydroprogesterone
P3a5a20a	5α-Pregnane-3α,20α-diol
P3b5a20a	5α-Pregnane-3β,20α-diol
P5b20a	5β,20α-Tetrahydroprogesterone
P3a5b20a	5β-Pregnane-3α,20α-diol
P3b5b20a	5β-Pregnane-3β,20α-diol
P3a5a17	17α-Hydroxyallopregnanolone
P3a5b17	17α-Hydroxypregnanolone
P3a5a17a20a	5α-Pregnane-3α,17α,20α-triol

P3b5a17a20a	5α-Pregnane-3β,17α,20α-triol
P3a5b17a20a	5β-Pregnane-3α,17α,20α-triol
A5a	5α-Androstane-3,17-dione
A3a5a	Androsterone
A3b5a	Epiandrosterone
A3a5b	Etiocholanolone
A3a5a17b	5α-Androstane-3α,17β-diol
A3b5a17b	5α-Androstane-3β,17β-diol
A3a5b17b	5α-Androstane-3α,17β-diol
F	Cortisol
E	Cortisone
B	Corticosterone
DOF	21-Deoxycortisol
DOC	11-Deoxycorticosterone
P3a5a11b21	3α,5α-Tetrahydrocorticosterone
P3a5b11b21	3α,5β-Tetrahydrocorticosterone
A211b	11β-Hydroxyandrostenedione
A3a5a11b	11β-Hydroxyandrosterone
A3b5a11b	11β-Hydroxyepiandrosterone
A3a5b11b	11β-Hydroxyetiocholanolone
PregC	Pregnenolone sulfate
Preg17C	17α-Hydroxypregnenolone sulfate
Preg20aC	20α-Dihydropregnenolone sulfate
DHEAC	DHEA sulfate
AdiolC	Androstenediol sulfate
AT16aC	5-Androstene-3β,16α,17β-triol sulfate
Prog17a20aC	Conjugated 17α,20α-dihydroxy-4-pregnen-3-one
Prog20aC	Conjugated 20α-dihydroprogesterone
TC	Conjugated testosterone
EpiTC	Conjugated epitestosterone
E1C	Estrone sulfate
E2C	Estradiol sulfate
E3C	Estriol sulfate
P3a5aC	Allopregnanolone sulfate
P3b5aC	Isopregnanolone sulfate
P3a5bC	Conjugated pregnanolone
P3b5bC	Conjugated epipregnanolone
P5a20aC	Conjugated 5α,20α-tetrahydroprogesterone
P3a5a20aC	Conjugated 5α-pregnane-3α,20α-diol
P3b5a20aC	Conjugated 5α-pregnane-3β,20α-diol
P5b20aC	Conjugated 5β,20α-tetrahydroprogesterone
P3a5b20aC	Conjugated 5β-pregnane-3α,20α-diol
P3b5b20aC	Conjugated 5β-pregnane-3β,20α-diol
P3a5a17C	17α-Hydroxyallopregnanolone sulfate
P3a5b17C	Conjugated 17α-hydroxypregnanolone
P3a5a17a20a	5α-Pregnane-3α,17α,20α-triol

P3b5a17a20a	5α-Pregnane-3β,17α,20α-triol
P3a5b17a20a	5β-Pregnane-3α,17α,20α-triol
A3a5aC	Androsterone sulfate
A3b5aC	Epiandrosterone sulfate
A3a5bC	Etiocholanolone sulfate
A3b5bC	Epietiocholanolone sulfate
AD3a5a17bC	Conjugated 5α-androstane-3α,17β-diol
AD3b5a17bC	Conjugated 5α-androstane-3β,17β-diol
AD3a5b17bC	Conjugated 5β-androstane-3α,17β-diol
AD3b5b17bC	Conjugated 5β-androstane-3β,17β-diol
P3a5a11b21C	Conjugated 3α,5α-tetrahydrocorticosterone
P3a5b11b21C	Conjugated 3α,5β-tetrahydrocorticosterone
A3a5a11bC	11β-Hydroxyandrosterone sulfate
A3b5a11bC	11β-Hydroxyepiandrosterone sulfate
A3a5b11bC	11β-Hydroxyetiocholanolone sulfate
